# Draft Genome Sequence of Phenol-Degrading Variovorax boronicumulans Strain c24

**DOI:** 10.1128/MRA.00597-20

**Published:** 2020-09-10

**Authors:** Fatma Azwani Abdul Aziz, Kenshi Suzuki, Koki Amano, Ryota Moriuchi, Hideo Dohra, Yosuke Tashiro, Hiroyuki Futamata

**Affiliations:** a Institute of Tropical Agriculture and Food Security, Universiti Putra Malaysia, Serdang, Selangor, Malaysia; b Graduate School of Science and Technology, Shizuoka University, Hamamatsu, Shizuoka, Japan; c Department of Applied Chemistry and Biochemical Engineering, Faculty of Engineering, Shizuoka University, Hamamatsu, Shizuoka, Japan; d Research Institute of Green Science and Technology, Shizuoka University, Shizuoka, Japan; University of Maryland School of Medicine

## Abstract

We report the draft genome sequence of Variovorax boronicumulans strain c24, which was isolated from a soil-inoculated chemostat culture amended with phenol as a sole carbon and energy source. The genome data will provide insights into phenol and other xenobiotic compound degradation mechanisms for bioremediation applications.

## ANNOUNCEMENT

The genus *Variovorax* belongs to the *Betaproteobacteria* and lives in soil and water and on plants (1, 2). A chemostat culture was amended with phenol as the sole carbon and energy source and trichloroethene (TCE)-contaminated aquifer soil as the inoculum (3). Strain c24 was isolated and purified aerobically from the chemostat culture using a plating method with MP medium (4) containing 0.5 mM phenol (MP0.5phe). The purity of strain c24 was confirmed by sequencing PCR-amplified 16S rRNA genes (3). Strain c24 is one of the strains exhibiting the highest affinity for TCE (3).

Genomic DNA of strain c24 cultivated in MP0.5phe medium was extracted by phenol-chloroform extraction (5) and fragmented using a Covaris M220 instrument according to the manufacturer’s protocol for a 550-bp fragment. A genomic library was constructed using a TruSeq DNA PCR-free library preparation kit (Illumina) according to the manufacturer’s instructions and sequenced on the Illumina MiSeq platform to generate 302-bp paired-end reads. The raw reads were cleaned up for quality using Trimmomatic v0.36 (6) by trimming adapter sequences, the 1 or 2 bases off the ends of the reads, low-quality ends with a quality score of less than 15, and reads of less than 150 bp. High-quality sequence fragments (1,071,488 paired-end reads, total of 491.7 Mb, and 70.5-fold coverage of the genome) were then assembled using SPAdes v.3.13.0 (7) with a default set of *k*-mer sizes and options (–careful, –only-assembler, and –cov-cutoff 20), and the contigs smaller than 200 bp were removed.

The draft genome sequence of strain c24 consists of 29 contigs with a total length of 6,973,636 bp, an *N*_50_ value of 1,080,070 bp, and a G+C content of 68.2%. Average nucleotide identity (ANI) analysis of the strain c24 whole genome using JSpeciesWS (8) showed the highest ANI value (96.8%) with the whole genome of Variovorax boronicumulans strain J1 (GenBank accession no. CP023284), suggesting that strain c24 belongs to *V. boronicumulans*. The numbers of protein-coding genes (CDS) and tRNA genes in the genome were predicted to be 6,493 and 63, respectively, by DFAST-core v1.2.0 (9). Sequencing coverages of the contigs were calculated by mapping high-quality reads to all contigs using the Burrows-Wheeler Aligner MEM algorithm (BWA-MEM) v0.7.12-r1039 (10) with default settings and analysis using QualiMap v2.2.1 (11). As a result, Contig20 (GenBank accession no. BKDI01000020.1), occupied by an rRNA gene operon (90%), shows 168.6-fold coverage, which is 2.4 times higher than the average coverage of the other contigs (69.9-fold). No other rRNA operons were found on extant contigs. This suggested that strain c24 contained two rRNA gene operons.

Strain c24 contained a multicomponent phenol hydroxylase (12–15), with *ortho*-cleavage metabolic pathways for catechol via catechol 1,2-dioxygenase (16). From the results of annotation, genes related to degradation of aromatic compounds (e.g., naphthalene, several kinds of benzoate compounds, functionally unknown monooxygenase, and dioxygenase) and heavy metal (e.g., Cu, Ag, Ni, As, and Co) transport systems were found.

### Data availability.

The complete genome sequence of Variovorax boronicumulans strain c24 has been deposited in DDBJ/ENA/GenBank under the accession no. BKDI00000000, and the raw sequencing reads have been deposited under the accession no. DRR189179.
